# SOMATOTROPIC SIGNALING IN HEALTH AND LONGEVITY

**DOI:** 10.1093/geroni/igac059.361

**Published:** 2022-12-20

**Authors:** Holly Brown-Borg

**Affiliations:** University of North Dakota, Grand Forks, North Dakota, United States

## Abstract

The endocrine system is highly integrated and regulates growth, reproduction, metabolism, and stress responses that influence aging. Reduced growth hormone (GH) signaling extends health and lifespan in part, by altering metabolism maintaining enhanced insulin sensitivity and defense mechanisms. Diet composition affects metabolism and GH status integrates these nutrient signals, modulating metabolic responses that result in age-related disease susceptibility. Two pathways affected by somatotropic signaling include methionine and lipid metabolism. GH appears to regulate oxidative defense and the methionine pathway via enzymes that affect S-adenosyl-methionine, glutathione, DNA methylation, and detoxification activities. We also have evidence that GH deficient mice escape fatty liver disease when fed high-fat diets. Together our work and others indicate that GH plays a significant role in an organism’s ability to respond to nutrients and cellular stressors by regulating factors that counter stress, modulating metabolic responsiveness to nutrients, and detoxification of endogenous and exogenous compounds.

